# Development and validation of a novel high performance liquid chromatography-coupled with Corona charged aerosol detector method for quantification of glucosamine in dietary supplements

**DOI:** 10.1371/journal.pone.0216039

**Published:** 2019-05-06

**Authors:** Chhavi Asthana, Gregory M. Peterson, Madhur Shastri, Rahul P. Patel

**Affiliations:** 1 School of Medicine, Division of Pharmacy, Faculty of Health, University of Tasmania, Hobart, Tasmania, Australia; 2 School of Health Sciences, Faculty of Health, University of Tasmania, Launceston, Tasmania, Australia; King Saud University, SAUDI ARABIA

## Abstract

**Introduction:**

Glucosamine dietary supplements are commonly used for the management of osteoarthritis (OA). However, clinical trials have reported varying outcomes with regard to joint function and disease progression. One of the possible reasons for variability in observed effects of glucosamine could be that, unlike prescription drugs, the quality of manufactured dietary supplements is not closely monitored in many countries. Therefore, there is the possibility that the actual amount of glucosamine present in a dietary supplement is different from that claimed on the label. The quality control of glucosamine supplements is further complicated by the unavailability of a simple and effective analytical method for the analysis of glucosamine. Therefore, the aim of this study was to develop a simple analytical method that could be easily adapted by the pharmaceutical industry for routine analysis of glucosamine.

**Aims:**

To develop a novel high-performance liquid chromatography (HPLC) method for the quantification of glucosamine, and determine the amount of glucosamine present in a sample of dietary supplements commercially available in Australia and India.

**Methods:**

Chromatographic separation of glucosamine was achieved using a zwitter-ionic hydrophilic interaction liquid chromatography column with a mobile phase consisting of 60% acetonitrile and 40% of 85 mM ammonium acetate, at a flow rate of 0.3 mL/min and column temperature 40°C. The developed method was validated for intra- and inter-day linearity, accuracy, precision, and reproducibility. The newly-developed method was subsequently used to analyse 12 glucosamine supplements.

**Results:**

The developed method was selective for glucosamine, which had a retention time of 5.9 min. The standard curve was linear with a correlation coefficient (r^2^) exceeding 0.99, over the range of 10–200 μg/mL for glucosamine. The relative standard deviations for intra- and inter-day accuracy, precision and reproducibility were all less than 4%. The amount of glucosamine determined in six Australian and six Indian glucosamine supplements ranged between 98.7–101.7% and 85.9–101.8% of the labelled values, respectively.

**Discussion:**

Unlike previous HPLC methods, this newly-developed HPLC technique does not require pre-derivatisation and can separate glucosamine from both hydrochloride and sulphate salts, and from other amino sugars, such as chondroitin sulphate present in dietary supplements. This simple and effective technique can be employed by analytical laboratories for the quality control of glucosamine dietary supplements.

**Conclusion:**

The current study has developed a new analytical technique using HPLC-Corona CAD, which can analyse underivatised glucosamine hydrochloride and sulphate within 6 minutes. Using the novel assay, we confirmed that unlike the tested Australian dietary supplements, only half of the tested Indian products had a glucosamine content within ±10% of what was claimed on the label.

## Introduction

Osteoarthritis (OA) is a degenerative joint disease commonly affecting the population aged 45 years and above [1]. In OA, the articular cartilage, which is composed of water and substances such as proteoglycans and collagen, is progressively degenerated. Amino sugars, such as glucosamine and chondroitin, are two main components of the glycosaminoglycan that constitutes proteoglycans [2, 3]. Therefore, glucosamine is commonly used as a dietary supplement for the management of OA; approximately 27% patients in America [4], 13% in Canada [5], 6.2% in Ireland [6], 12.2% in Korea [7], 8.8% in France, 14.3% in Spain [8], 26.2% in India [9] and 22% in Australia use glucosamine supplements for the management of their OA [10].

Randomised double-blinded placebo-controlled studies have indicated that glucosamine is effective in alleviating symptoms and improving joint movement [11] and can significantly delay disease progression [12] in patients with mild to moderate OA. On the other hand, some clinical studies have shown that glucosamine is neither effective in improving the symptoms associated with OA [13], nor useful in delaying the disease progression [14].

A number of reasons, such as varying dosage forms, salt forms of glucosamine [14, 15], treatment durations [11, 12], study end-points, dosage regimens [13, 14], concomitant use of analgesics [11], participants with various stages of disease progression [16, 17], and the use of either pharmaceutical grade or dietary supplement glucosamine [18–20] could be responsible for the observed discrepancies in reported clinical outcomes. One important reason for the observed inconsistent findings could be that the actual amount of glucosamine present in supplements varies from what is claimed on the label [18]. For example, a Canadian study investigated the amount of glucosamine present in 15 commercially available dietary supplements using high-performance liquid chromatography (HPLC). More than 93% of the tested supplements had a lower amount of glucosamine than what was claimed on the label, with the amount of glucosamine varying from 41 to 108% of the content stated [21].

HPLC and capillary electrophoresis coupled with ultraviolet (UV) detection are widely used analytical techniques for the analysis of pharmaceutical and dietary supplements. However, the major problem associated with the detection of glucosamine is that it does not contain the required UV chromophore. Therefore, pre-derivatisation of glucosamine with reagents is required to add a UV chromophore before analysis [22]. Similarly, glucosamine has no peculiar fluorophore and therefore the use of reducing agents are needed prior to its analysis using HPLC coupled with a fluorescence detector (FLD) [23, 24]. However, the process of derivatisation is laborious and time-consuming, and there is a possibility of analytical complications owing to the presence of by-products and excessive derivatising agent [25, 26].

The presence of a chromophore is not a prerequisite when HPLC is coupled with an evaporative light scattering detector (ELSD), mass spectrometry (MS) or refractive index (RI) detector. HPLC-ELSD and HPLC-MS have been used to quantify glucosamine present in a mixture of monosaccharides [27] and plasma [28], respectively. However, they have not been used or validated to quantify glucosamine in dietary supplements. Way et al. developed a reversed-phase ion-pair HPLC-RI technique for the quantification of glucosamine in one type of dietary supplement [29]. The main problem associated with the method developed by Way et al. was its inability to separate glucosamine from chondroitin sulphate, another amino sugar like glucosamine, commonly present in many glucosamine-containing dietary supplements. Studies have reported delayed disease progression of OA when glucosamine is used in combination with chondroitin sulphate, compared to glucosamine alone [12, 30].

Thus, there is a need to develop a simple analytical method that can effectively separate and quantify underivatised glucosamine and other ingredients, including structurally similar components, present in dietary supplements. Therefore, we aimed to develop and validate a simple HPLC method coupled with the Charged Aerosol Detector (CAD) to detect and quantify glucosamine. The developed method was validated and its application was demonstrated by analysing the glucosamine content of a sample of dietary supplements commercially available in Australia and India.

## Experimental

### HPLC instrumentation

A Dionex 3300 HPLC system (Thermo Fisher, California, USA) consisting of Dionex Ultimate 3000 RS pump with an internal degasser was used to pump and automatically mix mobile phase from separate chambers. Samples were auto-injected, and the temperature was constantly maintained with a Dionex Ultimate 3000 RS Autosampler. A Dionex Ultimate 3000 RS Column Compartment was employed to keep the column at a stable temperature. The analytes were detected using a Dionex Corona Ultra RS Charged Aerosol Detector (CAD). Chromeleon 7 Chromatography software was used to control the operations of the instrument and to run data acquisition.

### HPLC assay

A chromatographic separation was performed using a Zwitterion hydrophilic interaction liquid chromatography (ZIC-HILIC) column with a 200-Å pore size (SeQuant ZIC-HILIC, 150 mm × 4.6 mm × 5 μm, Merck Millipore, VIC, Australia).

For Corona CAD, nebuliser temperature was set at 25°C. Nitrogen gas was used as carrier gas at 2 L/minute flow rate to evaporate mobile phase and to produce particles of analytes. The data collection rate, power function and filter time constant were kept at 100 Hertz, 1 and 3 seconds, respectively. The temperature of the sample compartment was set at 10°C and the column compartment was either 25°C or 40°C. The injection volume was 5 μL.

### Method development

The stock solution of glucosamine (2 mg/mL) was prepared by dissolving 20 mg of glucosamine reference standard (glucosamine hydrochloride or glucosamine sulphate, Sigma Aldrich, NSW, Australia) in 10 mL of Milli-Q water and diluted with mobile phase to obtain a concentration of 80 μg/mL. For the method development, the mobile phase consisted of either 100, 85 or 80 mM of ammonium acetate (Sigma Aldrich, NSW, Australia) (mobile phase A) and various compositions of acetonitrile (50%, 60%, 65%, 75%, or 80%; Sigma Aldrich, NSW, Australia) (mobile phase B). The mobile phase A was prepared by dissolving 7.708 g, 6.55 g or 5.66 g of ammonium acetate in 1 L of Milli-Q water (Millipore Corporation, MA, United States) to prepare 100, 85 or 80 mM solutions, respectively. The mobile phase was filtered using a Millipore vacuum equipped with a 0.45 μm filter. The filtered mobile phase was degassed by ultrasonic bath (Sonorex RK-100, Bandelin Electronic, Berlin, Germany) for 10 minutes prior to its use. An isocratic elution of analytes was carried out using different compositions of mobile phase-A and mobile phase-B (40:60, 35:65, 30:70, 25:75 or 20:80 v/v). The mobile phase flow rate was set at either 0.3, 0.4 or 0.5 mL/minute.

When 100 mM ammonium acetate was employed, the composition of mobile phase B (acetonitrile) varied from 50–80%, the column temperature was either 25°C or 40°C and the flow rate varied from 0.3–0.5 mL/minute. When 85 mM or 80 mM ammonium acetate was employed, the composition of mobile phase B was 60%, the column temperature was 40°C and the flow rate was 0.3 mL/minute.

## HPLC assay performance

### Linearity

The stock solution (2 mg/mL) of glucosamine reference standard was serially diluted with Milli-Q water to obtain six different calibration standards containing 10, 20, 80, 160, and 200 μg/mL of glucosamine. The relationship between analyte concentration and the Corona-CAD response was determined by analysing glucosamine calibration standards. Calibration graphs were generated by plotting peak areas versus glucosamine concentrations and used to determine the linear regression coefficient (r^2^). Each sample was prepared in triplicate and analysed in triplicate.

### Accuracy, precision and reproducibility

Calibration standards with a low, medium and high concentration of glucosamine hydrochloride and glucosamine sulphate reference standard (10, 80 and 200 μg/mL) were used for the determination of accuracy, precision and reproducibility. Mean intra- and inter-day (3 consecutive days) accuracy values for the glucosamine peak (n = 6) were determined by the regression equation as follows: (observed concentration—expected concentration)/expected concentration × 100. Intra- and inter-day (3 consecutive days) precision values were investigated using peak areas with repeat analysis (n = 6) of glucosamine. Reproducibility was investigated by determining the mean intra- and inter-day (3 consecutive days) peak retention time of glucosamine. Each sample was prepared in triplicate and analysed in triplicate.

### HPLC system suitability

The system suitability was investigated using the tailing factor (T), theoretical plate number (N) (theoretical plate number was calculated using following equation: *N = 5*.*54 × (R*_*t*_*/W*_*0*.*5*_*)*^*2*^ */ l*, *where N = number of theoretical plates per meter*, *R*_*t*_ *= Retention time of glucosamine*, *W*_*0*.*5*_ *= peak width of glucosamine at half-height and l = length of HPLC column in meters*, the capacity factor (also known as retention factor; K), and resolution factor (Rs) for the glucosamine peak. The limit of detection and limit of quantification were investigated based on the signal-to-noise (S/N) ratio by establishing the minimum concentration at which the glucosamine could be reliably detected and quantified. Determination of the S/N ratio was performed by comparing the peak height of samples (5, 2.5, 1.25 μg/mL of glucosamine reference standard, n = 5) with those of blank samples (n = 5). The S/N ratio was established using the equation, *2H/h*, where *H* = peak height of each concentration (5, 2.5, 1.25 μg/mL) of glucosamine and *h* = the difference between the largest and smallest noise values of the blank sample. Both H and h values were measured between the same time width in chromatograms of the analyte (glucosamine reference standard) and the blank samples, respectively [31, 32].

## HPLC assay robustness

We investigated the robustness of the method by determining the effect of deliberate changes to pH of ammonium acetate (6.57 ± 0.2), composition of ammonium acetate (85 ± 2 mM), composition of acetonitrile (60% ± 2%) and the column temperature (40 ± 5°C) on peak area of glucosamine (80 μg/mL of glucosamine reference standard, n = 3). These parameters were selected as per the recommended guidelines [33, 34].

## HPLC assay application

The newly-developed method was applied to investigate the amount of glucosamine present in twelve different commercially available dietary supplements. Six supplements (capsule, tablet and liquid) were obtained from local pharmacies in Hobart, Tasmania, Australia and six supplements (tablet) were obtained from Bengaluru, Karnataka, India. The details of various brands of glucosamine supplements including brand name, type of formulation, salt form, amount claimed on the label, tablets were either film coated or uncoated are provided in Table 1. Three capsules and tablets were randomly selected and weighed individually, followed by calculation of the percent weight variation (%WV) using the following equation, %WV = (w/W) × 100, where ‘W’ is average weight of three randomly selected capsules or tablets and ‘w’ is the difference between the average weight and individual weight of capsule or tablet. The percentage weight variation calculated for capsules or tablets in each brand was less than 5% in all the brands as shown in Table 1, which is within the limit recommended by United States Pharmacopoeia (USP) [35].

**Table 1:** Brand name, type of formulation, salt form and amount claimed on the label of glucosamine supplements available in Australia and India.

Product No.	Brand name	Formulation	Glucosamine Salt	Claimed Amount	% Weight variation***
a	Wagner	Capsule^a^*	Glucosamine sulphate	750 mg	<2%
b	Nature’s Own	Tablet^b^*	Glucosamine hydrochloride	1500 mg	<1%
c	Nature’s Own	Liquid^c^*	Glucosamine hydrochloride	1500 mg	Not applicable
d	Blackmores Advanced	Tablet^d^*	Glucosamine sulphate^FC^	750 mg	<2%
e	Healthy Care	Capsule^e^*	Glucosamine hydrochloride	1000 mg	<1%
f	Swisse	Tablet^f^*	Glucosamine sulphate	1500 mg	<1%
g	Lubry-GM	Tablet^g^**	Glucosamine sulphate^FC^	750 mg	<1%
h	Jointace	Tablet^h^**	Glucosamine sulphate^FC^	750mg	<3%
i	Rejoint	Tablet^i^**	Glucosamine sulphate^FC^	500 mg	<3%
j	Apollo Pharmacy	Tablet^j^**	Glucosamine sulphate^FC^	1500mg	<1%
k	Cartigen Forte	Tablet^k^**	Glucosamine sulphate^FC^	750 mg	<2%
l	Lubrijoint Plus	Tablet^l^**	Glucosamine sulphate^FC^	750 mg	<2%

* = Australian Brand

** = Indian Brand

*** overall percent weight variation in each brand

^FC^ = Film coated tablets

^a^ = containing 1000 mg of glucosamine sulphate-potassium chloride complex equivalent to 754 mg of glucosamine sulphate with 140 mg of methylsulphonylmethane

^b^ = containing 1500 mg of glucosamine hydrochloride and 100 mg of chondroitin sulphate

^c^ = each 15 mL containing 1500 mg of glucosamine hydrochloride

^d^ = containing glucosamine sulphate-sodium chloride complex (942 mg) equivalent to 750 mg of glucosamine sulphate and 400 mg of chondroitin sulphate

^e^ = containing 1000 mg of glucosamine hydrochloride

^f^ = containing glucosamine sulphate-sodium chloride complex (1.88 mg) equivalent to 1500 mg of glucosamine sulphate with 500 mg *Zingiber officinale* or ginger equivalent to 2.5 mg gingerols

^g^ = containing 750 mg glucosamine sulphate-potassium chloride complex

^h^ = containing 750 mg of glucosamine sulphate-potassium chloride complex, equivalent to 446 mg of glucosamine with 250 mg of methylsulphonylmethane and 50 mg of diacerin

^i^ = containing 500 mg of glucosamine sulphate-sodium chloride complex with 400 mg chondroitin sulphate

^j^ = containing 1500 mg of glucosamine sulphate-potassium chloride complex

^k^ = containing 750 mg of glucosamine sulphate-potassium chloride complex with 100 mg chondroitin sulphate and 250 mg of methylsulphonylmethane

^l^ = containing 750 mg of glucosamine sulphate-potassium chloride complex, equivalent to 444 mg of glucosamine

## Extraction of glucosamine from capsules, tablets or liquid

Glucosamine capsules (n = 3) were weighed individually. The capsule shell was opened, and the content was emptied and weighed. The empty capsule shell was weighed, and its weight was added to its content weight to obtain a final weight value. This weight value was compared to the initial weight of the whole capsule to analyse any loss of capsule content during the process of emptying and weighing. The maximum percentage loss of capsule content was ≤0.02%. The capsule content containing either 750 mg or 1000 mg of glucosamine sulphate or hydrochloride was then mixed with either 15 or 20 mL of Milli-Q water to obtain a mixture expected to contain 50 mg/mL of glucosamine sulphate. Each sample was prepared in triplicate and analysed in triplicate.

Glucosamine tablet (n = 3), with or without film coating (please refer to Table 1) was placed in a clean porcelain mortar and crushed carefully, as finely as possible, using a pestle. The crushed tablet containing 500, 750, or 1500 mg of glucosamine hydrochloride or sulphate was then mixed with either 10, 15, or 30 mL of Milli-Q water to obtain a mixture expected to contain 50 mg/mL of glucosamine hydrochloride or sulphate. Each sample was prepared in triplicate and analysed in triplicate.

One mL of the liquid formulation (n = 3) containing 100 mg/mL of glucosamine hydrochloride was withdrawn from the amber-coloured glass bottle and mixed with 1 mL of Milli-Q water in a 15 mL plastic tube, to obtain a mixture expected to contain 50 mg/mL of glucosamine hydrochloride. Each sample was prepared in triplicate and analysed in triplicate.

The mixture (capsule, tablet or liquid) present in the plastic tube was centrifuged (CM-6MT, ELMI Ltd., Riga, Latvia) at 2300 rpm for 15 minutes. The supernatant (3 mL) was withdrawn using a 5 mL plastic syringe fitted with a 21G needle (Terumo Corporation, NSW, Australia). Following withdrawal of the sample, the needle was removed, and the syringe content was filtered using a 0.22 μm (25 mm) polyether sulphone (PES) syringe filter (Livingstone, NSW, Australia) to remove any particulate matter. The 10 μL aliquot of the collected filtrate was mixed with 990 μL of Milli-Q water to obtain a stock solution expected to contain a 500 μg/mL of glucosamine salt. The appropriate amount of this stock solution was then diluted with Milli-Q water to obtain a solution containing 80 μg/mL of glucosamine salt. Each sample was prepared in triplicate and analysed in triplicate.

## Extraction recovery

A known concentration of glucosamine sulphate reference standard solution (500 μg/mL) was spiked into the capsule content (n = 2, containing glucosamine sulphate) that was emptied into a tube. A known concentration of glucosamine hydrochloride reference standard solution (500 μg/mL) was spiked into the crushed tablet (n = 2, containing glucosamine hydrochloride) and into the liquid (n = 2, containing glucosamine hydrochloride) content. Extraction of glucosamine from the spiked capsule, tablet and liquid content was performed as described above and a mixture containing 50 mg/mL of either glucosamine sulphate or glucosamine hydrochloride was obtained.

The spiked mixture was then centrifuged, 3 mL supernatant was collected and then filtered using a syringe filter. The 10 μL aliquot of the filtered spiked solution was mixed with 990 μL of Milli-Q water to obtain a stock solution expected to contain 500 μg/mL of glucosamine salt. The stock solution was then diluted to obtain an expected glucosamine concentration of 80 μg/mL. Each sample was prepared in triplicate and analysed in triplicate.

## Stability of glucosamine

The stability of glucosamine hydrochloride and glucosamine sulphate was determined at 4°C and at room temperature for 48 hrs. The sample (10 mL) containing 200 μg/mL of glucosamine hydrochloride (n = 3) and 200 μg/mL of glucosamine sulphate (n = 3) was prepared and stored at 4°C and at room temperature. An aliquot (1 mL) was withdrawn at 0 (immediately after preparation), 2, 12, 24, 36 and 48 hours after the storage. The withdrawn samples were then analysed using the newly developed HPLC-Corona CAD method to determine the change in glucosamine concentration. Each sample was analysed in triplicate.

## Sample analysis

A calibration standard solution (n = 2) containing 80 μg/mL of either glucosamine hydrochloride or glucosamine sulphate reference standard was prepared. Glucosamine sample solutions, glucosamine spiked solutions and the reference standard were subjected to HPLC analysis. Each sample was prepared in triplicate and analysed in triplicate.

A calibration curve for both glucosamine hydrochloride and glucosamine sulphate reference standard was generated (10–200 μg/mL). The amount of glucosamine in each sample was calculated by comparing the peak area of glucosamine sample solution with the peak area of glucosamine reference standard. The extraction recovery of glucosamine was calculated by comparing the peak area of filtered glucosamine reference standard solution and glucosamine spiked solution with the peak area of glucosamine reference standard. Each sample was prepared in triplicate and analysed in triplicate.

## Results and discussion

### Effect of acetonitrile

**Fig 1:**
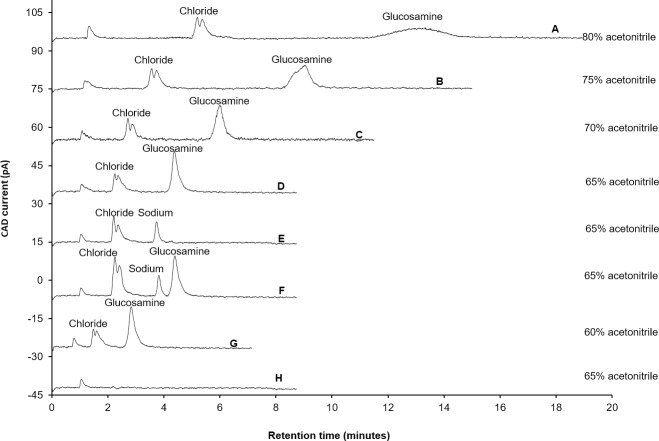
Influence of various compositions of acetonitrile on separation of glucosamine hydrochloride. Experimental conditions: ZIC-HILIC column (150, 4.6 mm), 200 Å, 5 μm; mobile phase containing various compositions of acetonitrile (as specified in chromatograms) and 100 mM ammonium acetate; column temperature was 25°C; detection with Corona-CAD; flow rate-0.5 mL/min; injection volume 5 μL.

Acetonitrile is an aprotic solvent and recommended as the preferred organic solvent for the ZIC-HILIC column because it has lower hydrogen binding ability or HILIC strength compared to other organic solvents, such as isopropanol, ethanol and methanol [36]. Acetonitrile has higher HILIC strength than acetone but has comparatively better separation ability [37]. In addition, acetonitrile is also recommended as an organic solvent with CAD due to its considerable volatility [38]. Therefore, only acetonitrile was employed as an organic modifier for the HILIC method development. The effect of different compositions of acetonitrile on separation, retention time, peak width and peak height of glucosamine hydrochloride was investigated by changing the composition of acetonitrile from 80 to 65%, (Fig 1A, 1B, 1C and 1D). The other parameters, such as composition of ammonium acetate (100 mM), flow rate (0.5 mL/min), column temperature (25°C) and CAD settings (data collection rate-100 Hertz, power function-1 and filter time constant-3 seconds), were kept constant. When the composition of acetonitrile was 80%, the retention time of glucosamine was 15 min (Fig 1A). The peak of glucosamine was broad with a peak width of 1.42 min and peak height of 2.855 pA. The retention time of glucosamine decreased from 15 min to 9 min, 6 min and 4.3 min when the acetonitrile composition was changed from 80 to 75% (Fig 1B), 70% (Fig 1C) and then 65% (Fig 1D), respectively. With 75% acetonitrile composition the peak height was increased from 2.855 pA to 8.956 pA, but the peak still appeared to be broad (peak width: 0.672 min). However, the peak height of glucosamine increased by more than 173% (peak height increased: 15.496 pA) and peak width decreased by 32% (peak width: 0.215min) when the acetonitrile composition was decreased from 75% to 65%. Using the same analytical conditions, sodium chloride (Fig 1E and 1F) was analysed to confirm that the peak eluting at 2.4 min, near to the solvent peak (Fig 1H), was chloride. The glucosamine peak eluted very near to the chloride peak and closer to the solvent front when the acetonitrile composition was decreased to 60% (Fig 1G). Therefore, further experiments were carried out using 65% acetonitrile composition.

As expected, reducing the composition of an organic modifier in the mobile phase decreased the retention time of glucosamine. In normal phase chromatography, water forms a pseudo-stationary layer over the polar stationary phase of the ZIC-HILIC column [39]. Glucosamine is polar in nature; therefore, it is likely to be immobilised in a water-rich pseudo-stationary layer [39, 40]. The elution rate of immobilised glucosamine depends on the hydrophilicity of the mobile phase. Therefore, reducing the acetonitrile composition from 80% to 65% weakened the interaction of glucosamine with the pseudo-stationary phase, resulting in a decreased retention time of glucosamine.

**Fig 2:**
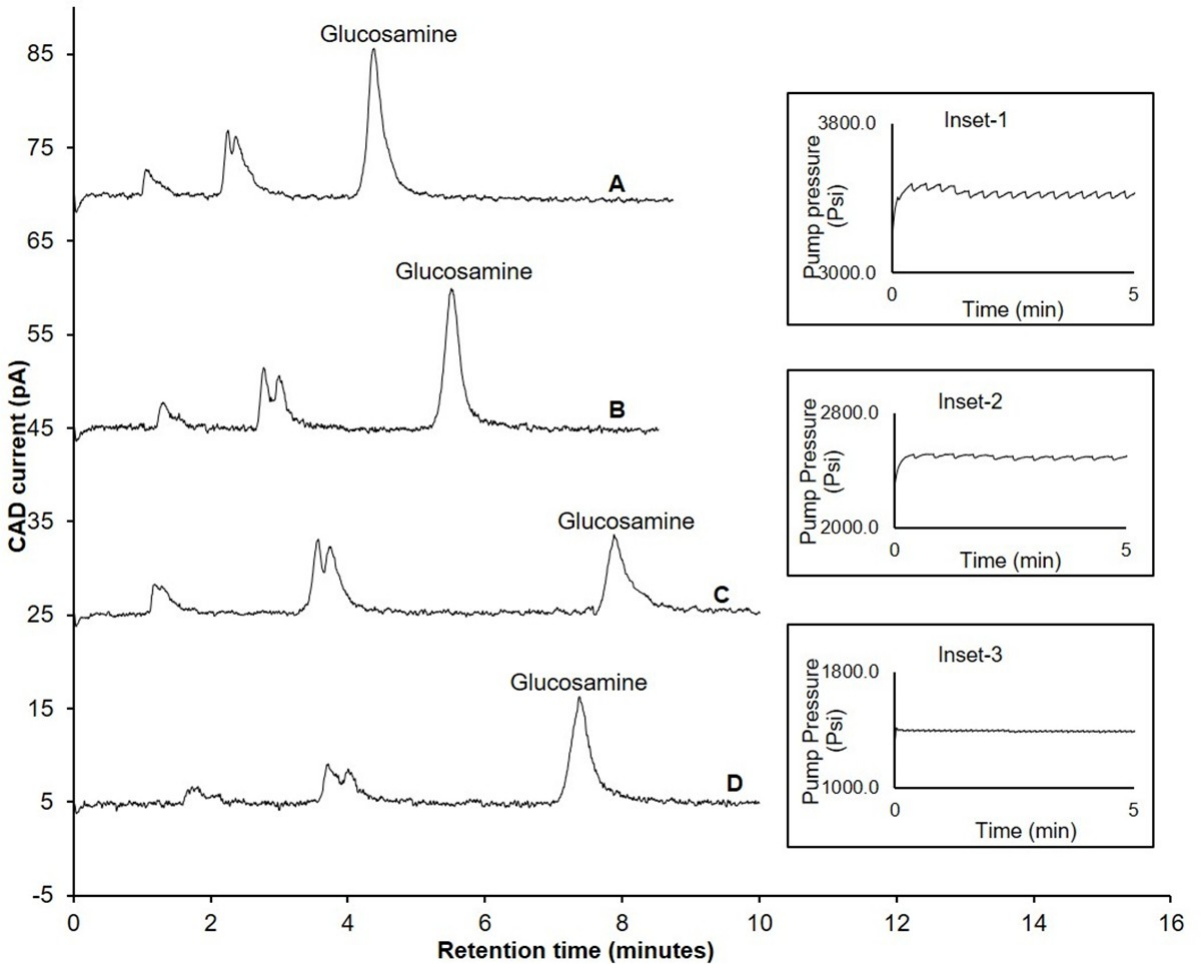
Influence of flow rate and temperature on backpressure and retention time of glucosamine hydrochloride. A) Retention time of glucosamine and backpressure of the column (Inset-1) when flow rate was 0.5 mL/min and column temperature was 25°C; B) Retention time of glucosamine when flow rate was 0.4 mL/min and column temperature was 25°C; C) Retention time of glucosamine and backpressure of the column (Inset-2) when flow rate was 0.3 mL/min and column temperature was 25°C; D) Retention time of glucosamine and backpressure of the column (Inset-3) when flow rate was 0.3 mL/min and column temperature was 40°C; Experimental conditions: ZIC-HILIC column (150, 4.6 mm), 200 Å, 5 μm; mobile phase containing 65% acetonitrile and 100 mM ammonium acetate; column temperature (as specified above); detection with Corona-CAD; flow rate- (as specified above); injection volume 5 μL.

Satisfactory separation was achieved when the composition of the mobile phase was 65% acetonitrile and 35% 100 mM ammonium acetate (Fig 2A). However, with the repeated injection (n = 10) of glucosamine, the column backpressure increased beyond the maximum allowed limit (5800 psi), resulting in either appearance of spikes during the chromatographic analysis (Fig 2 inset-1) or system error due to sudden termination of the pump operation. This could be due to two possible inter-related reasons; firstly, the use of a maximum recommended flow rate for the column (0.5 mL/min) and, secondly, a decrease in volatility of the mobile phase when the acetonitrile composition was decreased to 80% from 65%.

### Effect of flow rate and temperature on backpressure

To overcome the above-mentioned problem, the flow rate was first decreased from 0.5 mL/min to 0.4 mL/min, resulting in an increased retention time and acceptable peak symmetry (Asymmetry factor: 1.4) of (Fig 2B). However, the fluctuation in backpressure was still observed with repeated injections (n = 10) (Fig 2 inset-2) using 0.4 mL/min. Hence, the flow rate was decreased to a minimum recommended limit (0.3 mL/min). No fluctuation in retention time was observed at this flow rate with repeated injections of glucosamine hydrochloride (n = 10) (Fig 2 inset-3). As expected, the retention time of glucosamine was increased (Fig 2C), but the peak appeared to be tailing, compromising the peak symmetry (Asymmetry factor: 1.7). Therefore, we investigated the effect of temperature on glucosamine retention time and peak symmetry by changing the temperature of the column from 25°C to 40°C. At the latter temperature, glucosamine eluted at 7.9 min (Fig 2D), with acceptable peak symmetry (Asymmetry factor: 1.4).

**Fig 3:**
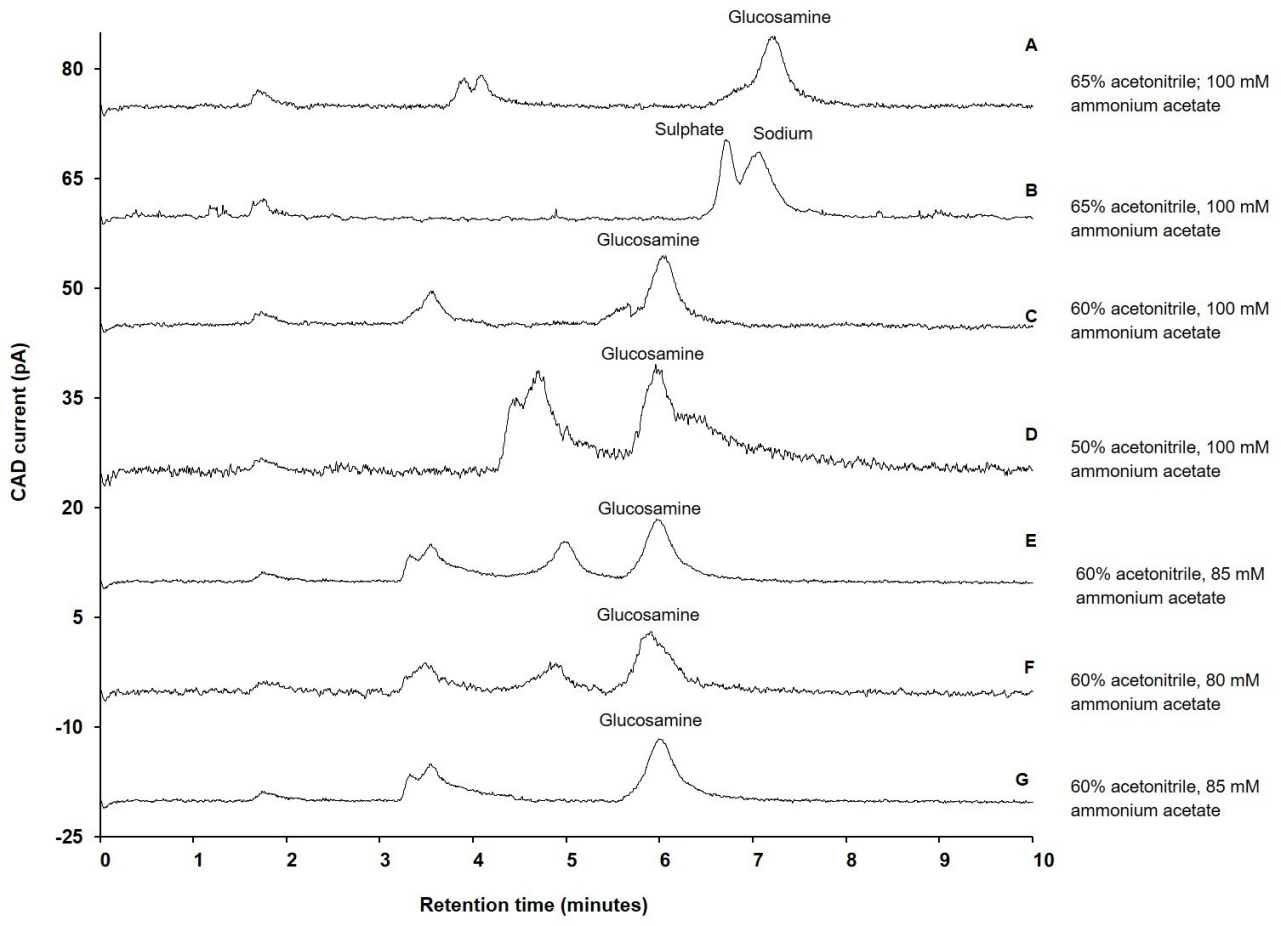
Influence of various composition of acetonitrile and ammonium acetate on separation of glucosamine. A) Separation of glucosamine sulphate when mobile phase contain 65% acetonitrile and 100 mM ammonium acetate; B) Separation of sodium sulphate when mobile phase contain 65% acetonitrile and 100 mM ammonium; C) Separation of glucosamine sulphate when mobile phase contain 60% acetonitrile and 100 mM ammonium acetate; Inset-1: Separation of glucosamine sulphate when mobile phase contain 50% acetonitrile and 100 mM ammonium acetate; D) Separation of glucosamine sulphate when mobile phase contained 50% acetonitrile and 100mM ammonium acetate; E) Separation of glucosamine sulphate when mobile phase contain 60% acetonitrile and 85 mM ammonium acetate; F) Separation of glucosamine sulphate when mobile phase contain 60% acetonitrile and 80 mM ammonium acetate; G) Separation of glucosamine hydrochloride when mobile phase contain 60% acetonitrile and 85 mM ammonium acetate; Experimental conditions: ZIC-HILIC column (150, 4.6 mm), 200 Å, 5 μm; mobile phase containing various composition of acetonitrile (as specified above) and ammonium acetate (as specified above); column temperature 40°C; detection with Corona-CAD; flow rate 0.3 mL/minute; injection volume 5 μL.

Attempts were made to separate the glucosamine sulphate using the same chromatographic conditions employed to separate glucosamine hydrochloride. The separation of glucosamine sulphate is shown in Fig 3A. It appeared that glucosamine co-eluted with another analyte, most likely sulphate, present in the sample. The composition of the co-eluted peak was confirmed by analysing sodium sulphate (Fig 3B). To overcome this problem, the composition of acetonitrile was changed from 65% to 60%, and then the retention time of glucosamine decreased to 5.9 min. However, the sulphate still co-eluted with glucosamine (Fig 3C). The acetonitrile composition was changed to 50%; however, it resulted in increased baseline noise (Fig 3D). This observed baseline noise could have been again due to an increase in viscosity and decrease in volatility of the mobile phase due to the decrease in composition of acetonitrile. Therefore, we investigated the effect of ammonium acetate on the separation of glucosamine sulphate.

### Effect of ammonium acetate concentration

The concentration of ammonium acetate was changed from 100 mM to 85 mM. At 85 mM concentration, sulphate was separated from glucosamine without affecting the retention time of the glucosamine peak (retention time: 5.9 min) (Fig 3E). When the ammonium acetate concentration was further decreased to 80 mM, no change in separation was observed but there was an increase in baseline noise (Fig 3F). Therefore, 85 mM ammonium acetate was selected for the analysis of the dietary supplements.

The co-elution of sulphate and glucosamine at 100 mM ammonium acetate could be due to the repulsive forces generated by sulphonate groups present at the terminal end of the stationary phase of the column being insufficient for early elution of sulphate ions, due to their interaction with positively charged ammonium ions supplied by 100 mM ammonium acetate [40]. However, at 85 mM ammonium acetate, the concentration of ammonium ions was decreased and availability of sulphonate groups increased resulting in increased repulsive forces required for early elution of the sulphate ions (Fig 3E). Using the same chromatographic conditions, glucosamine hydrochloride was successfully separated (Fig 3G).

## Assay performance

**Fig 4:**
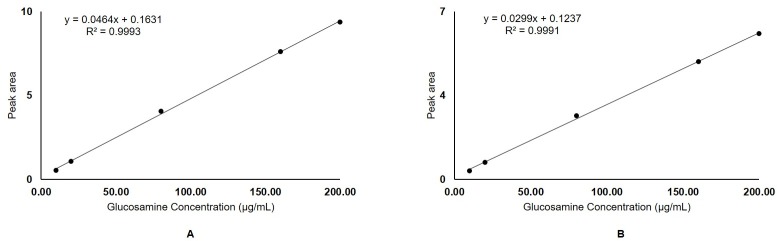
**Calibration curve with linear regression equations and correlation coefficients (r**^**2**^**) generated by plotting peak areas versus glucosamine concentrations (10–200** μ**g/mL) of A) Glucosamine hydrochloride reference standard; B) Glucosamine sulphate reference standard**.

The linear regression equation obtained for glucosamine hydrochloride and glucosamine sulphate was *y = 0*.*0464x + 0*.*1631* (Fig 4A) *and y = 0*.*0299x + 0*.*1237* (Fig 4B), respectively (where y is the peak area corresponding to the concentration, x of glucosamine). The linearity of the method estimated using correlation coefficient (r^2^) was found to be greater than 0.99 for glucosamine hydrochloride and glucosamine sulphate. The assay performance results are shown in Table 2. The mean inter- and intra-day accuracy and precision % relative standard deviation (%RSD) for each of the tested composition of glucosamine hydrochloride and glucosamine sulphate (10, 80 and 200 μg/mL) were found to be less than 4%. The mean inter- and intra-day reproducibility % RSD for each of the tested composition of glucosamine hydrochloride and glucosamine sulphate (10, 80 and 200 μg/mL) were found to be less than 3% and 2%, respectively.

**Table 2:** Mean inter- and intra-day accuracy, precision, reproducibility and linearity for glucosamine hydrochloride and glucosamine sulphate.

GH Concentr-ation (μg/mL)	Accuracy ^b^ (%RSD)		Precision ^c^ (%RSD)		Reproducibility ^d^ (%RSD)		Linearity	
	Inter-day ^a^	Intra-day	Inter-day ^a^	Intra-day	Inter-day ^a^	Intra-day	Slope / Intercept	r^2^
10	3.85	3.17	3.39	3.42	2.89	1.34	0.0464/ 0.1631	0.9993
80	3.62	2.88	2.94	2.16	2.92	1.51		
200	2.71	2.10	2.37	1.97	2.15	1.28		
GS Concentr-ation (μg/mL)								
10	3.52	3.10	3.21	3.27	2.97	1.42	0.0299/ 0.1237	0.9991
80	3.12	3.18	2.87	2.51	2.32	1.29		
200	2.13	2.12	2.04	2.09	2.11	1.19		

GH = Glucosamine hydrochloride; GS = Glucosamine sulphate

^a^ = mean %RSD of inter-day accuracy, precision or reproducibility determined for 5 consecutive days for the glucosamine peak (n = 6)

^b^ = determined by the regression equation as follows: (observed concentration—expected concentration)/expected concentration ×100

^c^ = determined using peak areas with repeat analysis

^d^ = determined using peak retention time of glucosamine with repeat analysis; r^2^ = correlation coefficient

The system suitability results are shown in Table 3. For both glucosamine hydrochloride and glucosamine sulphate the theoretical plate number for glucosamine was >13000, suggesting good efficacy and the resolution factor (Rs) was >1.9, indicating a good separation of glucosamine. The capacity factor (K) was ≥1.79 indicating the peak of glucosamine was well separated with respect to the void volume. The limit of detection and quantitation for glucosamine was found to be 1.25 and 5 μg/mL, respectively, for both glucosamine hydrochloride and glucosamine sulphate.

**Table 3:** Results of system suitability.

System Suitability Parameters	Observed Values	
	Glucosamine Hydrochloride	Glucosamine Sulphate
Theoretical plate number	13106	13111
Resolution factor	1.91	1.93
Capacity factor	1.87	1.79

The %RSD for peak area of glucosamine (80 μg/mL, n = 3) with change in 1) pH of ammonium acetate (6.27 or 6.87) was 1.52 and 1.75%; 2) concentration of ammonium acetate (83 or 87 mM) was 2.12 and 2.6%; 3) composition of acetonitrile (58 or 62%) was 2.29 and 2.8%; and 4) temperature (35 or 45°C) was 2.44 and 2.19%.

## Extraction recovery

The mean percentage extraction recovery of glucosamine hydrochloride and glucosamine sulphate standard was 100.2% and 100.3%, respectively. The percentage extraction recovery of glucosamine recovered from the spiked glucosamine formulations (capsule, tablet and liquid) that were prepared with filtration was found to be in the range of 99.3% to 101.9% (Table 4).

**Table 4:** Mean percentage recovery of glucosamine from spiked sample solutions after filtration.

Glucosamine source (n = 2)	Mean % recovery of glucosamine ± SD
Glucosamine hydrochloride standard	100.2 ± 0.03
Glucosamine sulphate standard	100.3 ± 0.85
Capsule^a^	100.5 ± 1.18
Tablet^b^	99.3 ± 0.139
Liquid^c^	101.9 ± 0.58

^a^ = product number-1

^b^ = product number-2

^c^ = product number-3, SD = standard deviation

It is important to investigate the effect of filtration on the recovery of pharmaceutical agents. Filtration could affect the concentration of a drug present in an admixture. For example, depending on the physicochemical properties of a drug and characteristics of the filter membrane, drug can be absorbed or adsorbed onto the filter membrane [41]. Drug molecules or excipients can interact with the filter membrane, resulting in drug loss [42]. In the current study, glucosamine tablets were crushed, dissolved and centrifuged. The supernatant before filtration contained hundreds of thousands of visible particles. The commercially available filters are designed to remove small particulate matter rather than suspension containing heavy particle load [42]. Therefore, depending on the filter membrane/area and applied pressure during filtration, the membrane could be ruptured resulting in contamination of the sample [43]. The samples were spiked with glucosamine standard to obtain extraction recovery, since a supplement may contain more or less than 100% glucosamine of the labelled amount. For example, if the extraction recovery of a glucosamine supplement was found to be 95%, when analysed without spiking its content with the reference standard, it would be difficult to determine whether the loss is due to filtration or the sample itself containing less than the labelled amount of glucosamine. Similarly, if a supplement actually contains 105% of the labelled amount, but its analysis indicates that the extraction recovery is 100%, then it is challenging to conclude whether this 5% loss in glucosamine content is due to loss during filtration. The extraction recovery of filtered glucosamine hydrochloride and glucosamine sulphate reference standard was found to be 100% and the extraction recovery of glucosamine from spiked sample was ±1% of the expected concentration.

No significant difference (p>0.05 with analysis of variance) was found between the mean extraction recovery of glucosamine extracted from spiked capsule, tablet or liquid samples and glucosamine hydrochloride and glucosamine sulphate reference standard, indicating that the polyethersulphone filter membrane is suitable and the method employed for extracting glucosamine from formulations was efficient.

## Stability of glucosamine

Although commercially available samples were analysed within 2 hours of their extraction and preparation, this study investigated the stability of glucosamine for up to 48 hours at 4°C and room temperature. The results are shown in Table 5. The concentration of glucosamine at time 0 hours was considered as 100%. Glucosamine concentration in glucosamine hydrochloride and sulfate was found to be greater than 99% when stored at 4 and room temperature for 48 hours.

**Table 5:** Stability of glucosamine stored at 4°C and room temperature up to 48 hours.

		Mean % of glucosamine hydrochloride remaining after each timepoint (SD)		Mean % of glucosamine sulphate remaining after each timepoint (SD)	
Time (hours)	Storage Temperature	4°C	Room Temperature	4°C	Room Temperature
2		99.9% (0.99)	99.7% (0.16)	102.0% (0.79)	99.5% (0.26)
12		100.7% (0.39)	100.4% (0.30)	100.0% (0.94)	100.1% (1.02)
24		99.6% (1.03)	102.9% (0.91)	100.2% (0.25)	100.3% (0.42)
36		99.4% (1.28)	99.2% (0.55)	100.9% (1.9)	100.1% (1.2)
48		100.2% (0.53)	100.5% (0.75))	101.9% (1.3)	100.1% (1.0)

The samples were stored up to 48 hours because at some instances, due to multiple number of samples, the analysis cannot be performed immediately after the sample preparation. Therefore, it is not uncommon to prepare and store the samples for a few days before analysis. In some cases, all the samples (for example, 100 samples) are prepared at once and kept for analysis in HPLC autosampler. If the runtime is 10 minutes, then the difference in the analysis time between the first and the last sample could be approximately 1000 minutes (16 hours). Therefore, change in the concentration, if there is any, could be either due to the degradation of the samples kept in the autosampler or could be due to the difference in the labelled amount and the actual amount of active ingredient present. Hence, to avoid such probability samples were stored for up to 48 hours. The results indicated that glucosamine was stable when kept at two different temperatures for 48 hours allowing the possibility of storing the samples before HPLC analysis.

## Method application

The newly-developed method was used to analyse 12 different glucosamine supplements containing either glucosamine hydrochloride or glucosamine sulphate with or without chondroitin sulphate. United States Pharmacopoeia/National Formulary suggests that the amount of glucosamine should not be less or more than 10% of the labelled amount of glucosamine [44]. The amount of glucosamine present in all the six tested supplements (100%) available in Australia was found to be within the limit of ± 5%, and only three out of six tested supplements (50%) available in India were found to be within the limit of ±10% of what was claimed on the label, while 50% of the tested Indian supplements contained less than 86% of the labelled amount of glucosamine (Table 6).

**Table 6:** Labelled and average amount of glucosamine observed in commercial preparations.

Product number	Formulation	Claimed Amount	Observed amount of glucosamine ± SD	Observed %glucosamine ± SD
a	Capsule*	750 mg	821.2 ± 58.3	105.6 ± 1.65
b	Tablet*	1500 mg	1444.4 ± 70.6	96.2 ± 4.7
c	Liquid*	1500 mg	1455 ± 18.3	97.9 ± 1.74
d	Tablet*	750 mg	795.5 ± 18.1	106.07 ± 2.42
e	Capsule*	1000 mg	1060.2 ± 24.8	106.02 ± 2.48
f	Tablet*	1500 mg	1572.1 ± 17.3	104.8 ± 1.15
g	Tablet**	750 mg	649.8 ± 17.7	84.4 ± 0.92
h	Tablet**	750 mg	757.1 ±12.8	100.9 ± 1.70
i	Tablet**	500 mg	509.2 ± 8.45	101.8 ± 1.69 69
j	Tablet**	1500 mg	1288.5 ± 4.67	85.9 ± 0.31
k	Tablet**	750 mg	631.5 ± 9.32	84.2 ± 1.24
l	Tablet**	750 mg	735.8 ± 8.35	98.1 ± 1.11

* = Australian Brand

** = Indian Brand

Glucosamine supplements are monitored under less stringent regulations than pharmaceutical products, although health agencies expect manufacturers to follow the Good Manufacturing Practice during the production of glucosamine dietary supplements [45]. This would also involve quantification testing to confirm that the finished product contains the declared amount of glucosamine.

One of the problems associated with routine analysis of glucosamine supplements through quality control in pharmaceutical industry is the lack of an easy and simple analytical method. Glucosamine possesses little UV chromophore and therefore CAD becomes the primary choice of detection. Unlike UV or photo diode-array (PDA) detector (method-1, 6, 7 and 8, Table 7) [21, 22, 46–48], the advantage of CAD is that the detection of an analyte does not depend on a UV chromophore but rather the mass of an analyte. Also, the limit of glucosamine detection with the current method was found to be 5 times greater than the previously reported method requiring derivatisation of glucosamine (method-1, Table 7) [21, 22]. In addition, unlike FLD (method 2, Table 7) [24], the use of CAD did not require pre-derivatisation of glucosamine. Unlike reversed phase columns employed in a previously reported method (method 3, Table 7) [28], ZIC-HILIC did not require pre-derivatisation of polar glucosamine to improve the retention time [49]. Derivatisation of glucosamine is not required when RI detector is used (method 4 and 5, Table 7). However, unlike RI detection, CAD is compatible with a wide range of organic modifiers and solvents. In addition, the limit of glucosamine detection was found to be 100 and 8 times greater than the previously reported HPLC-RI methods [29, 49]. The newly-developed method uses a small injection volume and therefore avoids problems associated with detector and column overload [50, 51]. Capillary isotachophoresis (cITP) coupled with conductivity detector (CD) was used to analyse underivatised glucosamine and chondroitin in dietary supplements (method 8, Table 7) [46]. For the effective separation of glucosamine, an acidic pH (pH = 4.7) using two different electrolytes must be maintained. For example, a decrease in electrolyte pH by 0.2 could result in coelution with glucosamine of other components, such as taste-masking agents like acesulfame potassium, and hydrolysed collagen. In addition, an increase in electrolyte pH would decrease the electrophoretic mobility of glucosamine along the capillary, resulting in increased elution time. On the other hand, with the newly-developed method, less than 2% RSD for peak areas of glucosamine was observed when the pH of mobile phase was changed from 6.57 to either 6.27 or 6.87, and no interference from other components was observed within this pH range. Both CAD and MS show comparable sensitivity in HILIC mode; however, unlike the MS detector, CAD does not require ionisation for detection [52], nor is any special training required to operate the instrument. It is also economical to use [53].

**Table 7:** Comparison of currently available methods for quantification of glucosamine with newly developed method.

Method No.	Analytical Technique/ Glucosamine salt^#^	Separati-on device (column or capillary	Detector	Derivatisation required	Retention time/ Total Run time (min)	Injection volume (μL)/ Minimum mass on column^a^ (μg)	LOD/LOQ (μg/mL)	Reference
Method-1	HPLC / GH	C18*	UV	Yes	29/40	100/0.125	0.63/1.25	[21, 22]
Method-2	HPLC/GH	C18*	FLD	Yes	9/14	10/1×10^−3^	0.030/0.1	[24]
Method-3	HPLC/GS	C18*	MS	Yes	7/21	5/0.5×10^−3^	0.035/0.1	[28]
Method-4	HPLC/GH	C18*	RI	No	NM/20	50/2.5	NM	[29]
Method-5	HPLC/GS	Amino*	RI	No	3.8/>8	20/0.4	NM	[49]
Method-6	CE/GH	Fused Silica**	PDA	Yes	≈2/3	0.02/ 3×10^−3^	≈18/NM	[48]
Method-7	CE/GH	Fused Silica**	PDA	Yes	11/15	0.02/ 2×10^−3^	1.3/NM	[47]
Method-8	cITP/GH	FEP** (2 types)	CD and UV	No	≈10.5/≈11	30/0.072	0.8/2.4	[46]
**Newly developed HPLC-CAD method**								
New Method	HPLC/GH and GS	ZIC-HILIC*	CAD	No	5.9/10	5/0.025	1.25/5	N/A

LOD = Limit of detection; LOQ = Limit of quantitation; HPLC = High performance liquid chromatography; GH = Glucosamine hydrochloride; GS = Glucosamine sulphate; CE = Capillary electrophoresis; cITP = Capillary isotachophoresis; UV = Ultra-violet; FLD = Fluorescence detector; MS = Mass spectrometry; RI = Refractive Index; PDA = Photo diode array detector; CD = Conductivity detector; CAD = Charged aerosol detector; Y = Yes; N = No; FEP = Fluorinated ethylene propylene; ZIC-HILIC = Zwitterion hydrophilic interaction liquid chromatography

^#^ = Glucosamine salt used for method development

* = Column

** = Capillary; NM = Not mentioned in the reference

^a^ = Minimum mass *(μg)* on column is the minimum quantifiable mass injected onto the column and calculated using equation- *Injection volume (μL) × LOQ or minimal concentration of standard curve of glucosamine (μg/μL)*; N/A = Not applicable

**Fig 5:**
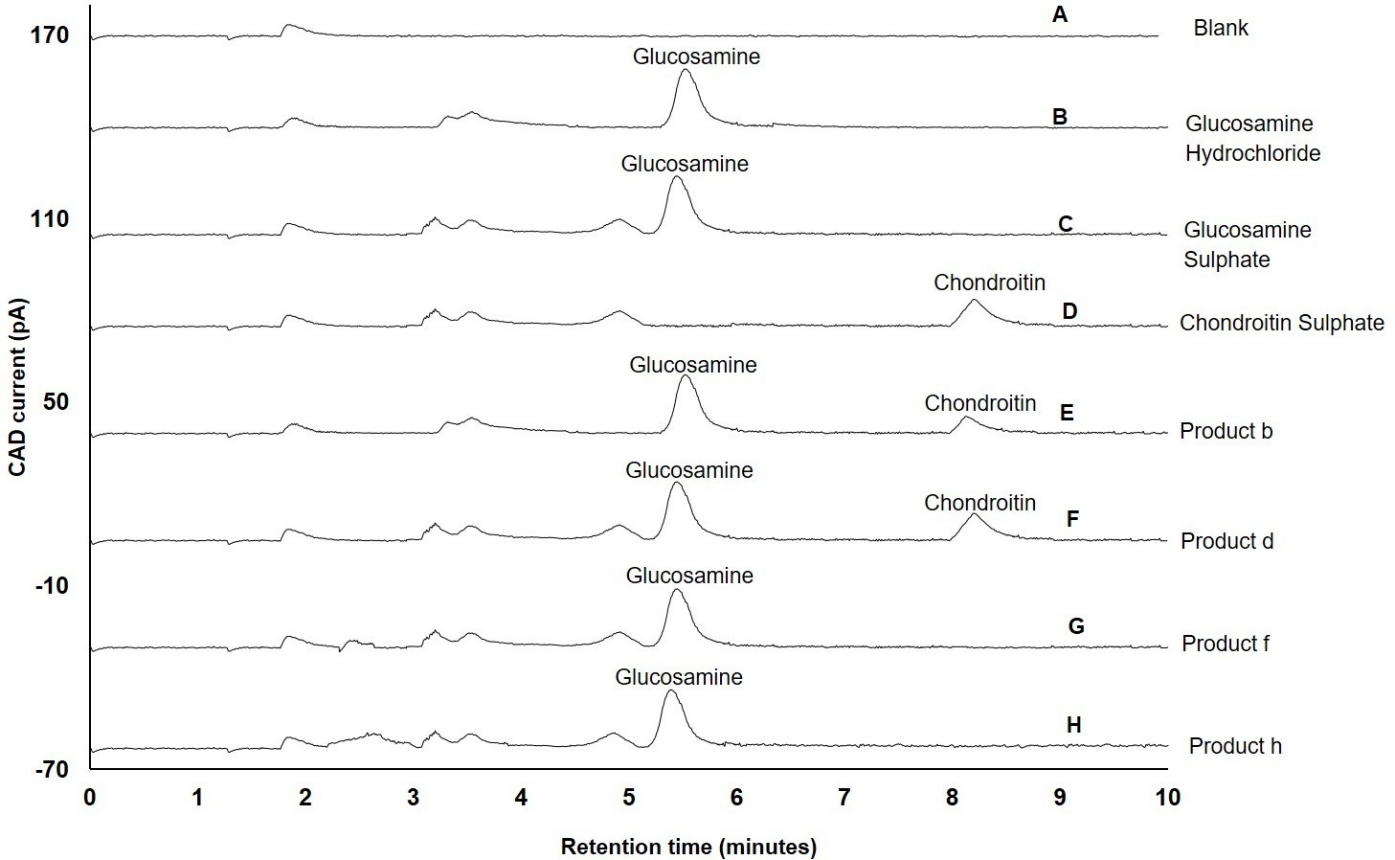
Application of newly-developed method for separation of glucosamine present in supplements. A) Blank (water); B) Glucosamine hydrochloride reference standard; C) Glucosamine sulphate reference standard; D) Chondroitin sulphate reference standard; E) Product-b containing glucosamine 1500 mg of glucosamine hydrochloride with chondroitin (100 mg) F) Product-d containing 750 mg of glucosamine sulphate with chondroitin (421 mg); G) Product-f containing glucosamine sulphate-sodium chloride complex (1.88 mg) equivalent to 1500 mg of glucosamine sulphate with 500 mg Zingiber officinale or ginger equivalent to 2.5 mg gingerols; H) Product-h containing 750 mg of glucosamine sulphate-potassium chloride complex, equivalent to 446 mg of glucosamine with 250 mg of methylsulphonylmethane and 50 mg of diacerin; Experimental conditions: ZIC-HILIC column (150, 4.6 mm), 200 Å, 5 μm; mobile phase containing 60% acetonitrile and 85 mM ammonium acetate; column temperature 40°C; detection with Corona-CAD; flow rate 0.3 mL/min; injection volume 5 μL.

In addition, other advantages offered by newly-developed method are a) glucosamine can be separated within 6 minutes, b) glucosamine can be separated from its two salt forms (Fig 5B and 5C) used in clinical practice, and c) glucosamine can be separated from other amino sugars, such as chondroitin sulphate, that are commonly present in glucosamine dietary supplements (Fig 5E and 5F). Apart from chondroitin sulphate, other ingredients present in glucosamine dietary supplements included 140 mg (product a) or 250 mg (product h and k) of methylsulphonylmethane (MSM), 500 mg Zingiber officinale or ginger and 2.5 mg gingerols (product f) and 50 mg of diacerein (product h), (please refer to Table 1).

In the current study, water was employed for the extraction of highly polar glucosamine from dietary supplements. Other ingredients present in dietary supplements in decreasing order of their water solubility were chondroitin sulphate > MSM > ginger or gingerols> diacerein [54–57]. Gingerols and diacerein are highly lipophilic, water insoluble and required to be dissolved in an inorganic solvent for extraction [56, 57]. Therefore, it is likely that during the extraction process diacerein and gingerols were precipitated and removed during filtration. Due to the degree of hydrophilic nature of chondroitin, MSM and certain water soluble components (phenolic, carbohydrates and soluble fibre) in ginger [58, 59] would have dissolved in the volume of water used for the extraction of glucosamine. However, no coeluting peaks of water-soluble ingredients with glucosamine peak were observed when product-a, f, h and k were analysed. The chromatograms of product f (comprising ginger/gingerols) and product-h (comprising MSM and diacerein) are shown in Fig 5G and 5H.

There are two limitations of the current study. First, the moisture content was not taken into account during the sample preparation step; however, this criterion is not recommended by USP as a part of routine quality control analysis of glucosamine tablets [44]. Second, the stability of glucosamine to physical forces was not investigated such as crushing during the sample preparation and centrifugation. However, this criterion is not recommended by USP; on the other hand, it recommends finely powdering the tablet to prepare the glucosamine sample for content analysis [44].

## Conclusion

The method developed is simple and selective for the detection and quantification of glucosamine. The method was successfully applied to 12 different commercially available glucosamine supplements, with a significant reduction in the run time and increased resolution compared with previously reported analytical methods. The selectivity and simplicity of this method allows its application in manufacturing for the identification and monitoring of batch-to-batch consistency of commercially available glucosamine products.
